# Personalized Approach in the Management of Difficult-to-Treat and Treatment-Resistant Depression With Second-Generation Antipsychotics: A Delphi Statement

**DOI:** 10.7759/cureus.52878

**Published:** 2024-01-24

**Authors:** Hansal Bhachech, Kamal Nath, Roop Sidana, Nilesh Shah, Rajesh Nagpal, R. Sathianathan, Ashutosh Kakkad, Krishnaprasad Korukonda

**Affiliations:** 1 Department of Psychiatry, Santvan Clinic, Ahmedabad, IND; 2 Department of Psychiatry, Silchar Medical College and Hospital, Silchar, IND; 3 Department of Psychiatry, Tekchand Sidana Memorial Psychiatric Hospital and Deaddiction Centre, Sriganganagar, IND; 4 Department of Psychiatry, Lokmanya Tilak Medical College, Sion, Mumbai, IND; 5 Department of Psychiatry, Manobal Clinic, New Delhi, IND; 6 Department of Psychiatry, Madras Memory Clinic, Chennai, IND; 7 Medical Services, Torrent Pharmaceuticals Limited, Ahmedabad, IND

**Keywords:** quetiapine, delphi, personalized care, aripiprazole, cariprazine, health knowledge attitudes practice, second generation antipsychotics, difficult to treat depression, treatment-resistant depression, mdd (major depressive disorder)

## Abstract

Background

Major depressive disorder (MDD) has many facets including mixed or atypical depression that requires personalized care to improve treatment-related outcomes. Second-generation antipsychotics (SGAs) offer complementary mechanisms for clinical roles in difficult-to-treat depression and treatment-resistant depression cases.

Aim/objective

To further delineate a consensus on the clinical positioning of SGAs for MDD, mixed, or atypical depression, a Knowledge Attitude Perception (KAP)-mediated Delphi Statement was planned.

Material/methods

A literature review for the definition, diagnosis, and management of MDD, mixed, and atypical depression as treatment-resistant depression (TRD) or difficult-to-treat depression (DTD) was conducted by a steering committee of academic and clinical experts (n=6) while developing a validated KAP questionnaire. Scientific statements as clinical recommendations were evolved using the Delphi methodology before building a clinical expert consensus with an online survey (n=24).

Results

Twenty-four psychiatrists highlighted DTD to offer a multidimensional approach to assess treatment strategies involving selective serotonin reuptake inhibitors (SSRIs) or SGAs, while ensuring symptom, functional, and quality of life (QoL) domain improvement for improved outcomes and remission rates. MDD cases with anxiety, anhedonia, comorbidities, and risk traits require personalized care with early induction of SGAs for severe cases or symptom persisters with functional impairment. Early augmentation with SGAs including aripiprazole or cariprazine can provide a favorable risk-benefit profile for clinical cases of MDD with or without the antecedent of mixed depression or personality disorder.

Conclusion

The literature review and KAP responses emphasize the importance of early identification for personalized care strategies with SGAs for DTD. Large-scale real-world evidence needs to evolve with due recognition of different phenotypes as TRD or DTD with partial or functional impairment to understand the impact of appropriate treatment pathways with SGAs.

## Introduction

Depressive disorders including major depressive disorder (MDD) and dysthymia remain an important clinical challenge while identifiable as the leading cause of disability worldwide. As per the World Health Organization (WHO), it affects 280 million people, the equivalent of 5% of the world's adult population [1]. As per the Global Burden of Disease Study 2019, depressive disorders accounted for 37.3%, the largest proportion of mental disorders disability-adjusted life years (DALYs) [2].

The Global Burden of Disease (GBD) study (2017) on the burden of mental disorders across the states of India suggests that 45.7 million adults suffer from depression, the equivalent of 3.3% of India's adult population. This study showed that the prevalence trend was significantly higher in females compared to males. Depressive disorders contributed the most to the total mental disorders DALYs (33.8%) in India [3]

MDD may cause significant functional impairment, which may require the use of psychotherapeutic, pharmacological, or neurostimulatory treatments. However, the major challenge is that a large proportion of patients have sub-optimal responses with residual symptoms that hamper the psychosocial well-being and harmony of the case despite the use of standard therapies. Achieving psychological well-being involves experiencing satisfaction, happiness, cognitive engagement, and a sense of meaning and purpose. Experts believe that there is also limited understanding of the comparative safety of antidepressant strategies in older adults, including risks of falls, cardiovascular risks, and risk of death with different agents used in trials. They also believe that augmentation may result in increased adverse effects and a higher likelihood of drug interactions [4]. Especially in elderly cases, there are safety concerns regarding the use of lithium or nortriptyline as approaches to treating treatment-resistant depression [5].

Although pharmacological therapy with antidepressants (AD) is a well-established and effective treatment for MDD, inadequate response to the initial AD treatment is seen in 30-60% of patients with MDD, and two consecutive AD therapies fail to elicit a response in an estimated 40% of patients. While there is no universally accepted definition of treatment-resistant depression (TRD), inadequate response to even one adequate trial of an antidepressant is a poor prognostic indicator, and the majority of cases have an increased risk of functional impairment and suicide, making it critical to identify effective treatments for individual patients [6,7,8].

The definition and consequent management of TRD has however been mired by several controversies taking into consideration the diverse pathobiological risk traits, neurostressors, comorbidities, and patient adherence that often confound the responses to initial-line standard of care and antidepressant therapy. Again, the omission, if not commission, of the key symptoms of depression that are often missed or underplayed with the incorporation of short-version diagnostic scales including HAM-D (Hamilton Rating Scale for Depression) or MADRS10 (Montgomery-Åsberg Depression Rating Scale) may underplay the low responder or remission rates that are often observed with the front-line therapy inducted in these cases of MDD. In most of these cases, pseudoresistance may be construed before augmentation of dose, duration, or any new drug class since these psychiatric drugs often need to cross the blood-brain barrier and bind to their target in the brain. Other factors include poor absorption of oral medication at the level of the gut endothelia, fast metabolism of medication by the liver, and poor blood-brain-barrier penetrance of medication. Certain treatments may exhibit a bell-shaped dose-response curve, wherein the dose-response seems to plateau even with high doses, often leading to pseudo-resistance [9].

In addition, the current definitions for nonresponse to treatment do not incorporate the substantial burden of commonly co-occurring symptoms, such as anxiety, irritability, and impairments in day-to-day functioning that patients with MDD often experience.

The focus on acute-phase treatment outcomes to define TRD has overlooked the chronic and recurrent nature of MDD and has led to calls for shifting the terminology toward "difficult-to-treat" depression, which may help incorporate response persistence, the burden of side effects, and the impact of therapies on daily functioning and quality of life while guiding the case for diagnosis as primary resistance requiring personalized approach with antidepressants offering multimodal actions or augmentation strategies as initial add-on therapy [10].

Data from different countries like North America [11], Sweden [12], and Hungary [13] have shown that patients with TRD have significantly higher all-cause mortality than other depressed patients. In addition, more treatment failures, and thus a greater degree of TRD, are associated with higher healthcare costs [14,15].

To further assess the scope, potential, and current contemporary practices regarding the role of different antidepressants for switching or combination, choice, and positioning of antipsychotics as augmenting agents in the management of treatment-resistant depression patients in real-world settings of India, a Knowledge, Attitude, Perception (KAP) survey was planned to be conducted to evolve a consensus on a personalized care approach in the management of MDD.

## Materials and methods

To define TRD and DTD, a consensus approach based on the Delphi method was created. This methodology provided operational criteria and algorithms that are relevant and applicable in Indian real-world scenarios.

**Figure 1:**
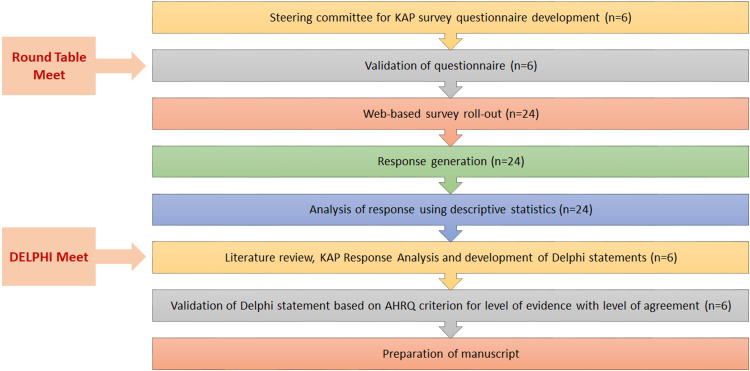
Study process flowchart AHRQ: Agency for Healthcare Research and Quality; KAP: Knowledge, Attitude, Perception

To discuss some of the pertinent issues in the management of TRD, with special emphasis on high-risk patients in the Indian context, a Knowledge, Attitude, Perception (KAP) survey questionnaire was developed. Six members of a multidisciplinary panel, including prominent practicing psychiatrists with national academic and/or clinical standing and relevant expertise in developing consensus statements, validated this questionnaire. The validated KAP survey was shared physically and electronically with twenty-four members of a multidisciplinary panel involving clinicians, academicians, and subject experts or researchers. Two rounds of digital meets were conducted between January 2023 and May 2023 for the development and validation of the KAP questionnaire and Delphi statements, respectively, by the steering committee (Figure 1).

The clinical specialists discussed the current state of the art and the major disagreements over the classification, definition, algorithmic approach, and management techniques, including augmentation tactics, during the meeting as they reviewed the pertinent literature. Clinical recommendations were evolved based on a successful problem-based clinical assessment method that is widely used in primary care while assessing the level of evidence as per the Agency of Healthcare Research and Quality (AHRQ) criteria and general expert opinion based on the clinical experience and insights shared by the panel with graded recommendations.

A systemic review of the literature was conducted to collate recent information or articles using PubMed, Google Scholar, and Cochrane Database of Systematic Reviews to search for the highest level of evidence (LoE) related to each recommendation from their date of initiation to March 2023. The relevant citations and their abstracts were reviewed by the panel who used the information before assigning graded recommendations based on the class of evidence (Class I, II, III, IV, and V) with Level of Agreement (LoA) or Consensus as on each recommendation: ‘strong’, indicating the top one-third of recommendations that had unanimous or almost unanimous (≥95%) agreement; ‘moderate’, indicating almost all the remaining two-thirds of recommendations, which had a substantial majority (61-94%); and ‘weak’, indicating the one recommendation that only reached the minimum consensus of 51%.

Each of the responses generated to the 10 questionnaires was given descriptive statistics for mean, median, and proportion analyses using MS Excel 2016 version (Microsoft Corporation, Redmond, USA). The response rate calculated as a percentage was also highlighted using descriptive statistics.

Compliance with ethics guidelines

Based on a clinical practice questionnaire, this study does not involve the participation of human subjects or patient data management. As such, this study was deemed exempt from requiring ethical approval.

## Results

Twenty-four practicing psychiatrists were approached to provide clinical insights on the contemporary positioning of TRD management using a structured knowledge, attitude, and perceptions (KAP)-based survey questionnaire. Within 30 days, responses were received from all 24 healthcare professionals (HCPs)from various geographical locations in India, which were collated and analyzed (Response Rate - 100%) (Table 1).

**Table 1:** Digital responses for KAP questionnaire from the web-based survey MDD: major depressive disorder; SNRI: serotonin-norepinephrine reuptake inhibitor; GAD: generalized anxiety disorder; MI: myocardial infarction; KAP: Knowledge, Attitude, Perception

No.	Structured questionnaire	Response	%
1.	What is the definition of treatment-resistant depression?	Resistance to a single antidepressant	12.5%
		Resistance to two antidepressant	83%
		Resistance to three antidepressant	4.5%
		None	0%
2.	What is the suggested duration of antidepressant use before classifying the case as treatment-resistant depression?	< 4 weeks	0%
		4 to 8 weeks	58.5%
		> 8 weeks	41.5%
		None	0%
3.	How many cases do not respond to >/= 2 antidepressants for diagnosis of MDD in your clinical practice?	10%	20.5%
		20%	45.5%
		30%	25%
		40%	4%
		>50%	0%
4.	Rate the choice of antidepressant in case of poorly controlled major depressive disorder with physical comorbidities including Ischemic heart disease or MI	Sertraline	50%
		Vortioxetine	16.66%
		Escitalopram	29.16%
		Dothiepin	4.16%
5.	Rate the choice of antidepressant for females presenting with GAD, MDD, and anhedonia	Sertraline	30.43%
		Escitalopram	21.73%
		Vortioxetine	30.43%
		Bupropion XL	4.34%
		Other:	8.69%
6.	Rate the choice of antidepressant for a 45y-old patient with MDD who has achieved partial remission with previous trials of sertraline, duloxetine, mirtazapine and finds difficulty in concentration, doing daily work with tasks taking more time than usual	Venlafaxine XL	21.73%
		Vortioxetine	52.17%
		Bupropion XL	4.34%
		Modafinil	4.34%
		Escitalopram	17.39%
7.	Rate the choice of antidepressant as add-on strategy for a 35-year-old patient with MDD presents with new depressive episode. The previous episodes were well treated with SNRI, however, this time the patient notes that this episode is more related to fatigue with significant lack of motivation to succeed at work and to socialize with friends	Venlafaxine XL	17.39%
		Vortioxetine	56.52%
		Bupropion XL	8.69%
		Sertraline	4.34%
		Escitalopram	13.04%
8.	Rate the first choice of antidepressant in treatment-naive MDD cases with anxiety features	Escitalopram	60.86%
		Vortioxetine	8.69%
		Sertraline	26.08%
		Mirtazapine	4.34%
9.	What is the choice of antipsychotics as an augmenting strategy in the management of drug or treatment-resistant depression?	Olanzapine	20.5%
		Quetiapine	12.5%
		Cariprazine	33.5%
		Aripiprazole	25.5%
		Others or Lithium	8%
10.	What is the dose-escalation strategy with cariprazine that is started at 1.5 mg for the management of treatment-resistant depression?	1.5 mg every week	66.5%
		1.5 mg every three days	16.5%
		0.75 mg every week	8%
		0.75 mg every three days	4.5%
		Slow titration:	4.5%

## Discussion

Treatment-resistant depression can be addressed through pharmacologic strategies such as augmentation, which involves adding an additional medication to an existing antidepressant, and switching, which entails replacing the current antidepressant with one from a different class [16]. The Sequenced Treatment Alternatives to Relieve Depression (STAR*D) trial demonstrated that bupropion substitution or augmentation was equally or more effective than alternative approaches [17,18]. In a randomized trial involving older adults, aripiprazole augmentation reduced depression more effectively than a placebo [19]. Aripiprazole or bupropion augmentation was marginally more beneficial than switching to bupropion in the Veterans Affairs Augmentation and Switching Treatments for Improving Depression Outcomes (VAST-D) trial [20], however, the low remission rates observed with the augmentation strategies including aripiprazole compared to ‘switching’ in the Optimizing Outcomes of Treatment-Resistant Depression in Older Adults (OPTIMUM) study [21] suggests the need for personalized care that may represent with differing symptomatology or functional impairments requiring a multi-interventional approach in most cases.

This was further highlighted in recent papers suggesting that incomplete response is not binary, but rather a continuum that ranges from partially responsive depression (PRD) to treatment-resistant depression (TRD) to multi-therapy resistant MDD (MTR-MDD) to refractory depression, which denotes no response to any of the currently recommended treatments [22]. The terms PRD and TRD are not optimal when it comes to their semantic and operational aspects.

In addition, the TRDs that refer to ≥2 antidepressants are fraught with challenges on the operationalization of non-response while offering little consideration on the clinical issues of treatment remission giving rise to ambiguities on treatment strategies involving drugs with differential mechanistics or class of compounds including SGAs. The Thase and Rush model of TRD classification as mild, moderate, or severe based on the involvement of ≥1 antidepressant with the duration and/or comorbidities further renders complexities towards a simplified approach to be adopted in the management of MDD and TRD [23].

In this line, the broader concepts of 'difficult-to-treat depression (DTD)' or 'suspected DTD' have been suggested to address pertinent issues of treatment resistance as symptom persisters despite ensuring adequate adherence to pharmacotherapy [10]. This concept overlaps with PRD and TRD but introduces a more flexible, multidimensional, and longitudinal definition with early adoption of scales for improved clinical staging, prognostication, and impact assessment on QoL involving Clinical Global Impression-Efficacy Index (CGI-EI)**, **Beck Depression Inventory (BDI)**, **and/or Sheehan Disability Scale​​​​​​​ (SDS) for overall assessment.

Even though several approaches have been developed, further strategies to ensure sustained response or continued remission for the management of TRD or DTD are required. Several guidelines outlining the treatment of MDD may help to direct practitioners in a logical, step-wise approach (Canadian Network for Mood and Anxiety Treatments​​​​​​​ (CANMAT), American Psychiatric Association (APA), National Institute for Health and Care Excellence (NICE)), however, specific guidelines for TRD and DTD are lacking and would ideally begin with the least invasive and intensive interventions which have the most evidence for efficacy in TRD [9].

There may be benefits to using different psychopharmacological therapies for adults with TRD. Antidepressants, lithium, triiodothyronine​​​​​​​ (T3), augmentation with SGAs, and more modern alternatives like ketamine and esketamine are some of these therapies. Combining antidepressants or changing from one antidepressant to another may work well. SGAs have proven to be clinically beneficial augmentation agents. Many have received FDA approval for MDD, and numerous studies have verified their effectiveness for the most part, especially when compared to changing to a different antidepressant. Nonetheless, it's crucial to remember that some SGAs, particularly the olanzapine-fluoxetine combination, can cause metabolic alterations. Lithium is probably more effective than combining a tricyclic antidepressant (TCA)with another antidepressant. It appears to be as effective as augmentation with SGAs, but its effectiveness is constrained by a low-dose approach that takes the agent's limited therapeutic index into account. Due to the paucity of data, T3 and lamotrigine have both been shown to be successful treatments for TRD and may be as effective as lithium. Recently, there have been encouraging results on the effectiveness of ketamine and esketamine for TRD; however, their differing routes of delivery may hinder treatment adherence [24,25,26,27].

**Figure 2:**
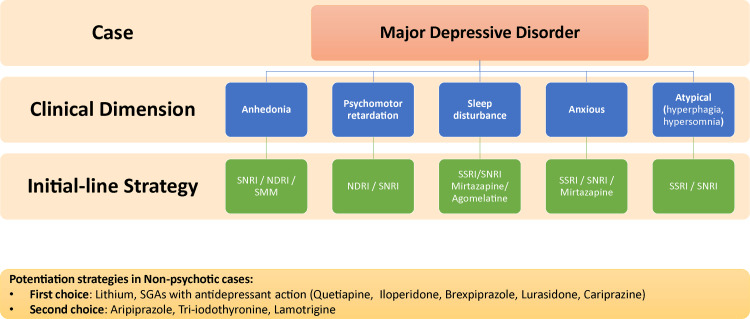
Treatment recommendations for specific clinical dimensions in MDD cases SSRI: selective serotonin reuptake inhibitor, SNRI: serotonin-norepinephrine reuptake inhibitor, NDRI: norepinephrine dopamine reuptake inhibitor, SMM: serotonin multimodal modulator, SGAs: second-generation antipsychotics

While more research is awaited before any conclusion on the differential positioning of these strategies for MDD, with incomplete response or TRD, a shared decision-making approach is the best course of action to determine which factors should be prioritized for each patient (Figure 2) [28].

Thirteen psychiatrists with TRD management clinical experience formed the South-East Asian (SEA) expert panel which has given a very simplified pathway for the management of adult MDD and PRD cases with Optimize, Switch, or Augment strategies [29]. However, overlooking the personalized care approach that may be relevant with augmentation strategies as an initial add-on instead of sequential therapy in such cases.

**Figure 3:**
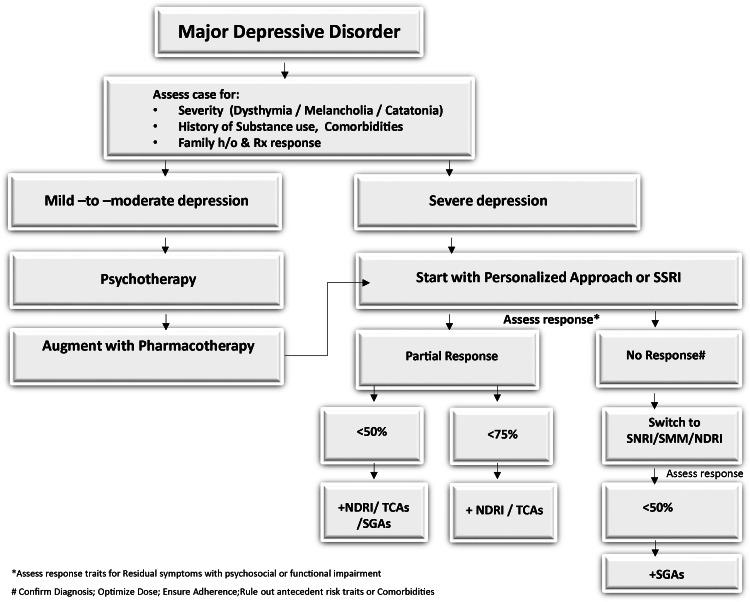
Clinical approach for MDD cases MDD: major depressive disorder; SSRI: selective serotonin reuptake inhibitor, SNRI: serotonin-norepinephrine reuptake inhibitor, NDRI: norepinephrine dopamine reuptake inhibitor, SMM: serotonin multimodal modulator, SGAs: second-generation antipsychotics; TCA: tricyclic antidepressants

An algorithmic approach based on clinical descriptors of MDD was evolved by the steering committee with the literature review, analyses, and evidence on the antidepressants and SGAs in the management of MDD showing incomplete response to at least one pharmacotherapy (Figure 3) [30].

The KAP survey aims to document the current contemporary clinical approach that is well-aligned with local guidance or statements regarding TRD management. In addition, the growing conviction with the local experience on the use of SGAs was well reflected in the choice of newer SGAs for the management of TRD while incorporating a personalized approach with SSRI/serotonin-norepinephrine reuptake inhibitor (SNRI)/norepinephrine-dopamine reuptake inhibitor (NDRI) for MDD.

Further clinical recommendations were evolved to foster diagnosis and management of MDD that is incompletely responsive or difficult to treat with at least one antidepressant drug exposure of adequate duration.

Clinical recommendations

Recommendation 1

The recommended outcome measure for evaluating treatment response (and remission) in MDD cases is the clinician-administered MADRS10. (LoE: Expert Opinion, LoA: 100%)

Recommendation 2

Currently, no biomarker has been proven to be effective in clinical practice or clinical trials to identify subjects with TRD patients who exhibit complete or incomplete non-responsiveness or to further stratify them. (LoE: Expert Opinion, LoA: 100%)

*Recommendation 3* 

Prior to making any clinical decisions on Switch or Augmentation strategies for cases currently on SSRI or SNRI therapy, comorbid personality disorders or other mental diseases should be ruled out. (LoE: Expert Opinion, LoA: 100%)

Recommendation 4

TRD suggesting an incomplete response to two or more antidepressants should involve medications of different mechanisms of action prescribed or used sequentially or as an augmented strategy. (LoE: Expert Opinion, LoA: 100%)

Recommendation 5

To determine DTD in clinical cases with significant symptoms and disease burden involving functional or psychosocial impairment that should be ascertained while ensuring minimal criteria of adequate dose and duration for at least four weeks despite personalized approach involving one or more pharmacotherapeutic agents. (LoE: Expert Opinion, LoA: 100%)

Recommendation 6

Augmentation strategies with SGAs (including cariprazine, aripiprazole, and quetiapine) remain feasible as add-on therapy in TRD and DTD cases receiving more than or equal to two antidepressants, especially in patients with personality traits. (LoE: II, LoA: 100%)

Recommendation 7

Cariprazine remains a feasible add-on strategy for the management of DTD, psychotic, and bipolar depression with metabolic traits or obesity, unlike other SGAs. (LoE: II, LoA: 100%)

Recommendation 8

Cariprazine at a dosage of 1.5-3 mg/d offers a low propensity for extrapyramidal symptoms​​​​​​​ (EPS)and akathisia in the management of TRD receiving ≥1 antidepressant showing incomplete response or persistent symptoms. (LoE: II, LoA: 83%)

Limitations

The KAP survey's findings and clinical recommendations suggest SGAs as front-line therapy for aspirational positioning in DTD, requiring further consolidation with dedicated survey studies​​​​​​.

## Conclusions

Treatment resistance comprises the trinity of establishing the correct psychiatric diagnosis, adequate treatment (in terms of dose and duration), and inadequate symptomatic response. It is commonly seen in many psychiatric disorders, including MDD, which may require augmentation strategies in most cases with drugs or drug classes with diversified mechanistics and better tolerability to ensure long remission and retention rates*.* Personalized care according to the risk traits or psychosocial variables may be apt to initiate therapies targeted towards the complete spectrum of symptom diversity while offering multimodal effects or actions.

Antipsychotics as an initial-line add-on or augmentation strategy serve a larger market for the treatment of difficult-to-treat depression, which is still under-optimized because of the current uncertainties surrounding diagnosis, prognosis, and pseudo-adherence used or adopted by HCPs in real-life settings in India. Atypical antipsychotics, including cariprazine, are the most extensively studied class of medication for antidepressant augmentation and are routinely prescribed in clinical practice for patients with TRD or DTD.

## References

[1] (2023). Depressive disorder - Key Facts. https://www.who.int/news-room/fact-sheets/detail/depression.

[2] (2020). Global burden of 369 diseases and injuries in 204 countries and territories, 1990-2019: a systematic analysis for the Global Burden of Disease Study 2019. Lancet.

[3] (2020). The burden of mental disorders across the states of India: the Global Burden of Disease Study 1990-2017. Lancet Psychiatry.

[4] Dixon LB, Holoshitz Y, Nossel I (2016). Treatment engagement of individuals experiencing mental illness: review and update. World Psychiatry.

[5] Köhler-Forsberg O, Larsen ER, Buttenschøn HN (2019). Effect of antidepressant switching between nortriptyline and escitalopram after a failed first antidepressant treatment among patients with major depressive disorder. Br J Psychiatry.

[6] Wiles N, Taylor A, Turner N (2018). Management of treatment-resistant depression in primary care: a mixed-methods study. Br J Gen Pract.

[7] Fife D, Reps J, Cepeda MS, Stang P, Blacketer M, Singh J (2018). Treatment resistant depression incidence estimates from studies of health insurance databases depend strongly on the details of the operating definition. Heliyon.

[8] Ng CH, Kato T, Han C (2019). Definition of treatment-resistant depression - Asia Pacific perspectives. J Affect Disord.

[9] Voineskos D, Daskalakis ZJ, Blumberger DM (2020). Management of treatment-resistant depression: challenges and strategies. Neuropsychiatr Dis Treat.

[10] McAllister-Williams RH, Arango C, Blier P (2020). The identification, assessment and management of difficult-to-treat depression: an international consensus statement. J Affect Disord.

[11] Li G, Fife D, Wang G (2019). All-cause mortality in patients with treatment-resistant depression: a cohort study in the US population. Ann Gen Psychiatry.

[12] Reutfors J, Andersson TM, Brenner P (2018). Mortality in treatment-resistant unipolar depression: a register-based cohort study in Sweden. J Affect Disord.

[13] Döme P, Kunovszki P, Takács P (2021). Clinical characteristics of treatment-resistant depression in adults in Hungary: real-world evidence from a 7-year-long retrospective data analysis. PLoS One.

[14] Johnston KM, Powell LC, Anderson IM, Szabo S, Cline S (2019). The burden of treatment-resistant depression: a systematic review of the economic and quality of life literature. J Affect Disord.

[15] Russell JM, Hawkins K, Ozminkowski RJ (2004). The cost consequences of treatment-resistant depression. J Clin Psychiatry.

[16] Ionescu DF, Rosenbaum JF, Alpert JE (2015). Pharmacological approaches to the challenge of treatment-resistant depression. Dialogues Clin Neurosci.

[17] Trivedi MH, Fava M, Wisniewski SR (2006). Medication augmentation after the failure of SSRIs for depression. N Engl J Med.

[18] Rush AJ, Trivedi MH, Wisniewski SR (2006). Bupropion-SR, sertraline, or venlafaxine-XR after failure of SSRIs for depression. N Engl J Med.

[19] Lenze EJ, Mulsant BH, Blumberger DM (2015). Efficacy, safety, and tolerability of augmentation pharmacotherapy with aripiprazole for treatment-resistant depression in late life: a randomised, double-blind, placebo-controlled trial. Lancet.

[20] Mohamed S, Johnson GR, Chen P (2017). Effect of antidepressant switching vs augmentation on remission among patients with major depressive disorder unresponsive to antidepressant treatment: the VAST-D randomized clinical trial. JAMA.

[21] Lenze EJ, Mulsant BH, Roose SP (2023). Antidepressant augmentation versus switch in treatment-resistant geriatric depression. N Engl J Med.

[22] McAllister-Williams RH, Christmas DM, Cleare AJ (2018). Multiple-therapy-resistant major depressive disorder: a clinically important concept. Br J Psychiatry.

[23] Fekadu A, Donocik JG, Cleare AJ (2018). Standardisation framework for the Maudsley staging method for treatment resistance in depression. BMC Psychiatry.

[24] Caldiroli A, Capuzzi E, Tagliabue I (2021). Augmentative pharmacological strategies in treatment-resistant major depression: a comprehensive review. Int J Mol Sci.

[25] Tundo A, de Filippis R, Proietti L (2015). Pharmacologic approaches to treatment resistant depression: evidences and personal experience. World J Psychiatry.

[26] Cowen PJ (2017). Backing into the future: pharmacological approaches to the management of resistant depression. Psychol Med.

[27] Haddad PM, Talbot PS, Anderson IM, McAllister-Williams RH (2015). Managing inadequate antidepressant response in depressive illness. Br Med Bull.

[28] Ruberto VL, Jha MK, Murrough JW (2020). Pharmacological treatments for patients with treatment-resistant depression. Pharmaceuticals (Basel).

[29] Tor PC, Amir N, Fam J (2022). A Southeast Asia consensus on the definition and management of treatment-resistant depression. Neuropsychiatr Dis Treat.

[30] Gautam S, Jain A, Gautam M, Vahia VN, Grover S (2017). Clinical practice guidelines for the management of depression. Indian J Psychiatry.

